# Inflammasomes in Sjögren’s Disease: Exploring the Therapeutic Value

**DOI:** 10.3390/ijms27146311

**Published:** 2026-07-15

**Authors:** Margherita Sisto, Sabrina Lisi

**Affiliations:** Department of Translational Biomedicine and Neuroscience (DiBraiN), Section of Human Anatomy and Histology, University of Bari “Aldo Moro”, 70124 Bari, Italy; sabrina.lisi@uniba.it

**Keywords:** Sjögren’s disease, inflammasomes, NLRP3, AIM2, inflammation

## Abstract

Inflammasomes arise from complex protein assembly mechanisms and play a fundamental role in managing inflammation and the innate immune response. The molecules that trigger inflammasome assembly and activation are molecules derived from pathogens or DNA fragments released following cellular damage. The phenomena resulting from inflammasome activation range from the activation of caspases, such as caspase-1, to the secretion of pro-inflammatory cytokines, to cellular death by apoptosis or pyroptosis. Various diseases have been linked to aberrant inflammasome activation, including several autoimmune diseases, leading scientists to direct experiments toward identifying the mechanisms responsible for aberrant inflammasome activation so as to develop new therapeutic strategies. In this review, we summarize the assembly mechanisms and involvement of two specific inflammasomes, NLRP3 and AIM2, in autoimmune Sjögren’s disease (SjD). NLRP3 and AIM2 aberrant activations appear to be involved in the exacerbation of inflammation, which becomes chronic, leading to dry mouth and dry eye and to an increased risk of developing B-cell non-Hodgkin’s lymphoma in these patients. Understanding how different inflammasomes contribute to the pathogenesis of SjD could be fundamental to a better understanding of the complex molecular mechanisms underlying this disease.

## 1. Introduction

The inflammasome is an intracellular complex of oligomers that detects and responds to PAMPs (Pathogen-Associated Molecular Patterns) and DAMPs (damage-associated molecular patterns). PAMPs and DAMPs are signal molecules recognized by the innate immune system via pattern recognition receptors. Specifically, PAMPs are derived from microbic lipopolysaccharide (LPS) or viral RNA, while DAMPs are endogenous molecules released by damaged or necrotic cells [1]. Four main types of inflammasomes have been identified: AIM2 (Absent in melanoma 2), NLRP1 (NOD-, leucine-rich repeat (LRR)-, and pyrin domain-containing protein 1), NLRP3, and the NLRP4 inflammasome. Assembly of the inflammasomes involves the cleavage and activation of caspases and pro-inflammatory cytokines [1].

Sjögren’s disease (SjD) is a chronic systemic autoimmune disease characterized by lymphocytic infiltration in the exocrine glands. Primary SjD (pSjD) is characterized by dysfunction and destruction of salivary glands (SGs) and lacrimal glands associated with chronic lymphoepithelial lesions, not related to clinical manifestations affecting other organs, which occur in secondary SjD [2]. pSjD features a broad clinical spectrum extending from disease confined to exocrine glands up to systemic disease and B-cell lymphoma development [2]. Although an association between inflammasome activation in peripheral blood cells and disease complexity in SjD has been reported, little is known about inflammasome activation in exocrine glands and its underlying mechanisms. Dysregulated inflammasome activation is responsible for several autoinflammatory diseases associated with high levels of proinflammatory cytokine secretion, including SjD. Significant progress has been made in understanding inflammasome assembly and activation, although molecular data are still lacking, making this research a rapidly developing field. This review describes the current structural and biomolecular understanding of the activation mechanisms of NLRP3 and AIM2 inflammasomes, focusing on discoveries regarding the role of these molecular complexes in SjD. The purpose of this study was to review literature sources regarding the structure and assembly of inflammasomes and how they contribute to SjD. Articles were selected from PubMed for review if their content included (1) inflammasomes and (2) SjD. The concept that glandular epithelia play a critical role in the activation of NLRP3 and AIM2 inflammasomes, which are involved in the chronic inflammation that characterizes this autoimmune disease, is explored and highlighted in this review.

## 2. Summary of the Main Pathogenetic Features of Sjögren’s Disease

SjD is a complex systemic autoimmune condition primarily characterized by chronic inflammation of the SGs and lacrimal glands and a widespread plethora of extra-glandular features and immunological anomalies [3]. SjD is a recognized model of multifactorial diseases triggered by the overlap of environmental, hormonal, and genetic events [4]. The disease involves many organs and systems, with possibly severe manifestations and increased risk of B-cell lymphoma such as non-Hodgkin’s lymphoma of the mucosa-associated lymphoid tissue (MALT-NHL). Unfortunately, SjD is a heterogeneous and disabling condition that negatively affects the patient’s quality of life, with enhancemed morbidity and mortality compared to the worldwide population [5]. During the 15th International Symposium on Sjögren’s disease (Rome, 2022), the complexity of the disease, which currently leads to the use of the term “syndrome,” was highlighted, emphasizing the multiple clinical aspects and the variety of long-term outcomes [6]. Factors originating outside the body, playing a role in the development of SjD, include viral infections, female hormones, solvents, and inorganic chemical agents [7,8]. The genetic factors associated with SjD were specifically explored in the initial genome-wide association study of the disease conducted in 2013, which pointed out a significant link between SjD and *HLA-DQB1* [9,10]. Moreover, those findings emphasized polymorphisms in *IRF5*, *STAT4*, *BLK (B-lymphoid kinase)*, *IL12A*, *TNIP1*, and *CXCR5* genes, which appeared to be linked with a greater susceptibility to SjD [11]. The products of these genes are involved in important pathophysiological steps of the disease. Since then, large-scale genetic and epigenetic findings have evidenced interplay between SjD and genes involved in both innate and adaptive immune systems. Epigenetic modifications such as DNA hypomethylation, histone acetylation, and microRNA expression also concur with SjD pathophysiology [12].

In the last decade, different studies have considered the epithelial cells derived from the SGs (SGECs) not to be “innocent bystander” targets of cellular and humoral autoreactivity against cellular antigens [13]. In fact, the epithelia, considered classically as physical barriers, are now recognized as a dynamic tissue in which the cells are actively involved in the constitutive or inducible expression of numerous factors and implicated in innate and acquired immunological responses [14]. The SGECs in the glandular lesions of SjD release molecules that promote persistent inflammatory reactions and direct lymphocyte chemoattraction. This finding supports the proposed description of SjD as an “autoimmune epithelitis” [15,16]. Immunohistochemical data on inflamed SG tissues derived from SjD patients have suggested that ductal and acinar SGECs produce high levels of several immunoactive molecules that are known to mediate lymphoid cell homing, antigen presentation, and the amplification of epithelial–immune cell interactions [16,17]. Therefore, SGECs next to sites of strong inflammation have been shown to express high levels of major histocompatibility complex (MHC) proteins class I (HLA-ABC) and class II (HLA-DR) molecules, CD54/ICAM1, CD106/VCAM, and E-selectin adhesion molecules [18]. Numerous investigations in SjD patients have shown an increased epithelial production of pro-inflammatory cytokines. In particular, researchers have shown a significant release of IL-1β, IL-6, IFN-γ, and TNF-α, not only by infiltrating lymphocytes but also by ductal cells in SjD SGs [17,19]. IL-1α, IL-1β, IL-8, TGF-β, and granulocyte macrophage-colony-stimulating factor (GM-CSF) productions were revealed in both acinar and ductal cells. Furthermore, the SGECs can act as antigen-presenting cells, producing lymphoid chemokines, an increased expression of the CD40/CD40L complex, adhesion molecules, and the release of cytokines and BAFF [14]. This demonstrates their potential role in the recruitment of immune cells, including T, B, and dendritic cells, in the severe inflammatory lesions of SjD SGs and in the production of lymphoid tissue. In addition, SjD SGECs have recently been shown to express high constitutive TLR protein expression, suggesting intrinsic activation of epithelial cells in SjD and further supporting the role of this type of tissue in the pathogenesis of the disorder [20,21].

In the last few years, research has been focused on the involvement of multiprotein complexes called ‘inflammasomes’ within the autoimmune picture, such as SjD. Crucial, for example, is the NLRP3 inflammasome serving to amplify tissue inflammation through the involvement of interleukin-1β and IL-18, which are overexpressed in SjD. A deregulated NLRP3 inflammasome activation occurs in the serous acini of salivary and lacrimal glands prone to SjD [22]. These findings may provide novel biomarkers and new therapeutic targets for the management of SjD patients with adverse outcomes.

## 3. The NLRP3 Inflammasome Complex: An Overview

The inflammasomes are cytosolic supramolecular complexes constituted by a signaling machinery composed of three components: sensor, adaptor and effector [23]. This platform can induce the proteolytic maturation of proinflammatory cytokines and pyroptotic cell death in response to endogenous stimuli known as DAMPs, which originate from the host, and to exogenous stimuli known as PAMPs, such as bacterial toxins [24,25]. Other triggers include reactive oxygen species, oxidized mitochondrial DNA, and lysosomal disruption [24].

One of the factors most extensively studied is the NLRP3 inflammasome, a tripartite protein composed of NLRP3, ASC (apoptosis-associated speck-like protein containing a caspase recruitment domain), and caspase-1 [26,27].

**Figure 1:**
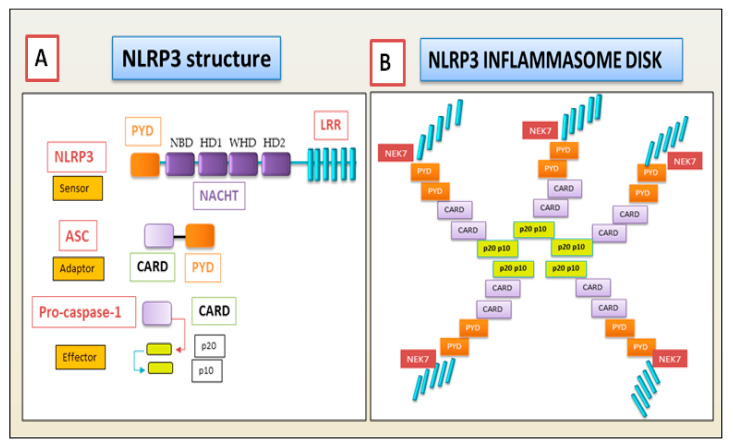
NLRP3 inflammasome machinery (**A**): NLRP3 structure: NLRP3 is constituted by three regions: an N-terminal PYD, 12 repeat LRR domains at the C-terminal end, and a central NACHT domain. The NACHT domain can be subdivided into four subdomains, marked as NBD, HD1, WHD, and HD2. The adaptor ASC is composed of an N-terminal PYD, and a C-terminal CARD. Caspase-1 consists of an N-terminal CARD, a large catalytic subunit (p20), and a C-terminal small catalytic subunit (p10). (**B**): NLRP3 inflammasome disk: schematic representation of the assembly of mature NLRP3. NLRP3 interacts with ASC via its PYD, and ASC interacts with caspase-1 through its CARD. This leads to the cleavage of the p20 and p10 segments of pro-caspase-1, activating caspase-1. (ASC, apoptosis-associated speck-like protein containing a caspase recruitment domain; CARD, C-terminal caspase recruitment domain; HD1, helical domain 1; LRR, leucine-rich repeat; NBD, nucleotide-binding domain; NEK7, NIMA-related kinase 7; PYD, pyrin domain; WHD, winged helix domain).

The sensor NLRP3 (also known as cryopyrin) is formed by three parts: an amino (N)-terminal pyrin domain (PYD), a carboxy (C)-terminal LRR, and a central NBD-containing ATPase domain named NACHT [23,24]. PYDs typically engage in homotypic interactions to modulate downstream signaling pathways. LRR domains are primarily responsible for facilitating protein–protein interactions, and the LRR segment of NLRP3 has also been associated with preserving its stability. The NACHT domain has ATPase activity that is necessary for NLRP3 self-association following its activation, leading to self-interactions between PYDs that, subsequently, recruit ASC, which serves as the adaptor [24]. The ASC adaptor features an N-terminal PYD and a C-terminal caspase recruitment domain (CARD). It is brought to the clustered PYDs of the oligomerized NLRP3 proteins through homotypic PYD-PYD interactions, resulting in the creation of a prion-like ASC filament [24]. These nucleated filaments assemble the C-terminal CARDs of ASC, which act as a platform to recruit caspase-1, the effector [26]. Caspase-1 is composed of an N-terminal CARD, a major catalytic unit referred to as “p20”, and a smaller catalytic unit at the C-terminal known as “p10” [23]. The CARDs of caspase-1 engage with the aggregate ASC CARDs and experience analogous nucleated filament development [28,29]. These filaments promote proximity-induced dimerization of the p20 and p10 catalytic subunits of caspase-1 and lead to self-cleavage at the junction between p20 and p10, rendering caspase-1 completely proteolytically active [30,31]. While there is no biophysical constraint on the variety of stoichiometries these proteins can adopt, the estimated relative abundance of ASC to caspase-1 in cells is about 1:3.5 according to quantitative Western blot analysis [30]. When activated, caspase-1 cleaves and activates gasdermin D (GSDMD), which triggers pyroptosis, and transforms the pro-cytokines in the IL-1 family into mature proinflammatory cytokines, crucial for immune response regulation [31]. A detailed overview of NLRP3 structure and mechanism of assembly is shown in Figure 1.

### 3.1. Mechanism of NLRP3 Inflammasome Activation: Canonical Pathway

The activation of the NLRP3 inflammasome is a multifaceted mechanism triggered by various stimuli such as microorganisms, environmental factors, and endogenous stress signals. Three mechanisms are known to trigger the NLRP3 inflammasome, including canonical, non-canonical, and alternative pathways [32]. Primarily, in the resting cells, the NLRP3 inflammasome is mostly found in an inactive state that becomes activated upon stimulation and subsequently assembles into a large cytosolic complex. In this scenario, classical canonical activation relies on two independent signal steps: priming (signal 1) and assembly (signal 2) [1]. The priming step of inflammasome activation requires the recognition of an NLRP3 activator that induces full activation and inflammasome formation. NLRP3 is activated by bacterial, viral, and fungal infections, while in sterile inflammation it is mediated by the recognition of various PAMPs or DAMPs that engage pattern-recognition receptors (PRRs) such as Toll-like receptors (TLRs) or nucleotide-binding oligomerization domain-containing protein 2 (NOD2), or through cytokines such as tumor necrosis factor (TNF), or else exposure to environmental irritants [33]. The trigger of the priming signal by TLR or TNF through MyD88 leads to NF-kB activation, and it is commonly associated with ‘‘danger signals”, such as bacterial LPS, which are found at their surfaces and can bind to TLR4. To prepare NLRP3 for the subsequent assembly phase, priming signals also entail either preventing degradation or releasing self-inhibition by inducing post-translational modifications (PTMs) [34]. These activators are able to induce cellular stress that is subsequently detected by NLRP3 [35]. The precise mechanisms by which NLRP3 detects cellular stress and the specific pathways that lead to its activation and the formation of the inflammasome have yet to be fully clarified. It is believed that several upstream signals contribute to this process, many of which can occur simultaneously, such as the efflux of potassium ions (K^+^) or chloride ions (Cl^−^), fluctuations in calcium ions (Ca^2+^), lysosomal disruption, mitochondrial dysfunction, metabolic alterations, and disassembly of the trans-Golgi [36]. While there is a wealth of information available regarding the upstream signaling events, many of the pathways are interconnected and overlapping, and the findings can sometimes be contradictory.

**Figure 2:**
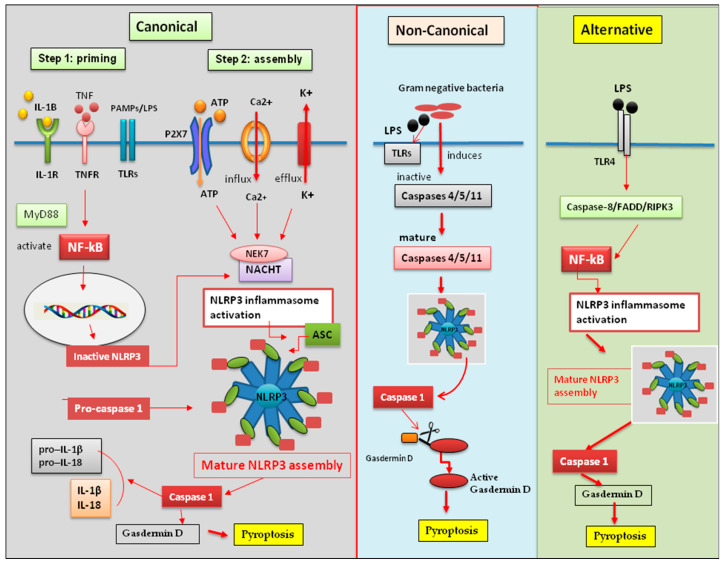
Mechanism of NLRP3 inflammasome activation. The NLRP3 inflammasome can be activated through canonical, non-canonical, and alternative pathways. The canonical activation of the NLRP3 inflammasome is composed of two signals. Step 1, the priming signal, is induced by cytokines like TLR4 or TNF, and then it activates the NF-κB pathway, triggering the upregulation of NLRP3 protein, pro-caspase-1, pro-IL-1β, and pro-IL-18 expression. During step 2, the assembly signal, numerous PAMPs or DAMPs promote NLRP3 inflammasome assembly, triggering pro-caspase-1 self-cleavage and activation. Extracellular ATP and K^+^ efflux through the P2X_7_ receptor and Cl^−^ efflux promote the NLRP3 inflammasome activation. Active caspase-1 cleaves the immature cytokines, such as pro-IL-1β and pro-IL-18, to produce active cytokines IL-1β and IL-18, respectively. It also cleaves gasdermin D and releases its N-terminal domain, determining the pyroptosis process and the release of the mature inflammatory cytokines. Non-canonical NLRP3 inflammasome activation occurs in response to cytosolic LPS induced by caspase-4/5/11. Induction of NLRP3 inflammasome activation is triggered by K^+^ efflux that leads to activation of gasdermin D. Alternative NLRP3 inflammasome activation requires a TLR4 activation-dependent signal in monocytes through the RIP1-FADD-caspase-8 pathway. (FADD, Fas-associated protein with death domain; NF-κB—nuclear factor kappa-light-chain-enhancer of activated B cells; IL-1β, interleukin-1β; IL-18, interleukin-18; RIP, receptor-interacting protein; TLR, Toll-like receptor).

Following the priming phase, the second step is oligomerization, where the NLRP3 inflammasome forms an active and mature multiprotein complex constituted by NLRP3, ASC, and pro-caspase-1. The mature complex is activated to process and release IL-1β and IL-18 [37]. NLRP3 activation involves the assembly in oligomer cages, where the pyrin domain (PYD) is protected inside to maintain an inactive state, and an auto-inhibited conformation. Upon stimulation, the dynein adapter histone deacetylase 6 (HDAC6) drives the microtubules transport, and the Microtubule Organizing Center (MTOC) is essential for functional inflammasome assembly [38]. Inside the MTOC, NLRP3 interacts with NIMA (never in mitosis gene A)-related kinase 7, also known as NEK7, to create a heterodimer, resulting in a structural change in NLRP3 that reorganizes the oligomer and reveals the PYD. Following this, NLRP3 attracts ASC through a PYD–PYD interaction, prompting ASC oligomerization, which subsequently enables ASC to recruit pro-caspase-1 through CARD–CARD binding. Ultimately, all three elements are combined to create a complete and extremely active NLRP3 inflammasome [39]. At this stage, active caspase-1 cuts the cytokine precursors pro-IL-1β and pro-IL-18 to generate the active cytokines IL-1β and IL-18. It also cleaves gasdermin D (GSDMD), releasing its N-terminal domain, which moves to the cell membrane to create pores, resulting in the release and pyroptosis of mature inflammatory cytokines [40]. Figure 2 illustrates the canonical pathway of NLRP3 activation.

### 3.2. NLRP3 Non-Canonical Activation and Alternative Pathway

The non-canonical inflammasome activation varies considerably from the classical mechanism of NLRP3 activation, yet both result in cell lysis and the release of proinflammatory cytokines [41]. In the non-canonical pathway (Figure 2), the activation of caspase-4/5 takes place in humans and caspase-11 in mice. Cytosolic LPS can activate the noncanonical inflammasome independently of the priming step [32]. Additional research indicated that the conserved lipid A region of LPS is accountable for non-canonical inflammasome activation [32]. Lipid A is recognized directly by the CARD of caspase-4/5/11, resulting in its oligomerization, which is followed by the cleavage of the pore-forming protein gasdermin D at the linker connecting the N-terminal and C-terminal domains by active caspases [32]. The released N-terminal domain of GSDMD interacts with the plasma membrane and creates membrane pores with an inner diameter of 10–14 nm, enabling potassium efflux, pyroptosis, and subsequent activation of the NLRP3 inflammasome [42]. Consequently, caspase-4/5/11 do not cleave interleukins; instead, they trigger pyroptosis, and the ensuing potassium efflux-induced NLRP3 inflammasome activation is accountable for caspase-1 activation and IL-1β secretion [43]. These data indicate the relationships between the canonical and non-canonical pathways involved in inflammasome activation [43]. The alternative pathway only exists in monocytes, in which TLR4 recognizes extracellular LPS and induces NLRP3 activation and cytokine maturation through the caspase-8/FADD/RIPK3 signaling pathway, but neither apoptosis-associated speck formation nor pyroptosis is induced (Figure 2) [24].

## 4. The NLRP3 Inflammasome in Sjögren’s Disease

The NLRP3 inflammasome is a prominent regulator of innate immunity, and its aberrant activation exacerbates pathological injury by promoting the release of inflammatory factors, cellular pyroptosis, and fibrosis [44]. The NLRP3 inflammasome is closely associated with several systemic diseases; growing scientific findings confirm its critical involvement in various autoimmune diseases, such as rheumatoid arthritis (RA), systemic lupus erythematosus (SLE), systemic sclerosis, and ankylosing spondylitis, where its activation appears to be correlated with worsening disease activity [45]. Recent years have seen increasing evidence of the implication of the NLRP3 inflammasome in SjD [46]. Studies have demonstrated that the NLRP3 inflammasome and its downstream factors are upregulated in peripheral blood mononuclear cells isolated from SjD patients; these levels are correlated with disease activity, contributing to xerostomia and glandular dysfunction [46,47,48]. Baldini et al. first demonstrated that NLRP3 and caspase-1 mRNA expression was enhanced in labial SG tissue specimens from patients with SjD compared to those with sicca syndrome [47]. Deregulated NLRP3 inflammasome activation also occurs in the lacrimal glands of SjD, and tear fluid from SjD patients revealed higher mRNA expression of NLRP3 and caspase-1 compared to healthy controls [47]. Recently, various pathogen-derived and endogenous molecules, including nucleic acids released following tissue injury, have been shown to induce inflammasome activation [32,48,49]. These undegraded DNAs serve as DAMPs to activate the NLRP3 inflammasome [50]. High cell-free DNA levels and impaired DNase1 activity have been detected in the serum of patients suffering from severe SjD. SjD patients manifest aberrant clearance of apoptotic cells by phagocytes and impaired serum DNase1-mediated degradation of necrotic cell remnants; these factors are associated with increased amounts of circulating nucleosomes and cell-free DNA [51]. Furthermore, findings reveal that peripheral monocytes and SG-infiltrating macrophages in SjD patients exhibit NLRP3 inflammasome activation. This is particularly evident in severe cases and is likely induced by widespread accumulation of extranuclear DNA in serum, cells, and tissues, where deficient DNA degradation by deoxyribonucleases plays a major role [51]. Compelling evidence indicates that inflammatory processes in the salivary lesions of SjD patients can also be attributed to the activation of the P2X7R-inflammasome axis, a component of the NLRP3 machinery [52]. Interestingly, the involvement of the P2X7R-inflammasome axis was confirmed in a large cohort of SjD patients: the P2X7R–inflammasome complex was significantly overexpressed compared to controls, particularly in patients positive for anti-Ro/SSA with higher focus scores in salivary gland biopsies [53]. The expression of both P2X7R and inflammasome components was significantly higher in pSjD gland specimens, paralleled by increased levels of mature IL-18 in saliva samples compared to non-SjD sicca syndrome and healthy subjects. These data are correlated with anti-Ro/SSA positivity and lymphocytic sialadenitis focus scores, establishing a link between P2X7R signaling and pSjD exocrinopathy [52]. Moreover, elevated expression of P2X7R, NLRP3, caspase-1, and IL-18 has been observed in SjD patients developing MALT-NHL [52]. Consequently, the P2X7R/NLRP3 axis-driven inflammation contributes to glandular epithelial injury and secretory dysfunction [46]. From this perspective, it is hypothesized that the P2X7R/NLRP3 complex might be a hallmark of distinct immunohistopathological features and the varying complexity of lymphocytic infiltrate in SjD, ultimately contributing to the degenerative processes leading to MALT-NHL development [3,54]. In addition, NLRP3 activation is triggered by mitochondrial dysfunction and DNA damage-associated DAMPs, which amplify IL-1β-driven acinar cell death and fibrosis; whereas, pharmacological inhibition of NLRP3 partially restores salivary flow in animal models [55]. DAMP-induced NLRP3 activation in obstructive and chronic sialadenitis has also been reported to induce macrophage recruitment, polarization, and glandular tissue remodeling [46]. Taken together, these data confirm that NLRP3 inflammasome activation exacerbates disease activity. Accordingly, the inhibition of upstream and downstream factors of the NLRP3 inflammasome may be a promising therapeutic strategy.

## 5. Discovery and Structure of the AIM2 Inflammasome

**Figure 3:**
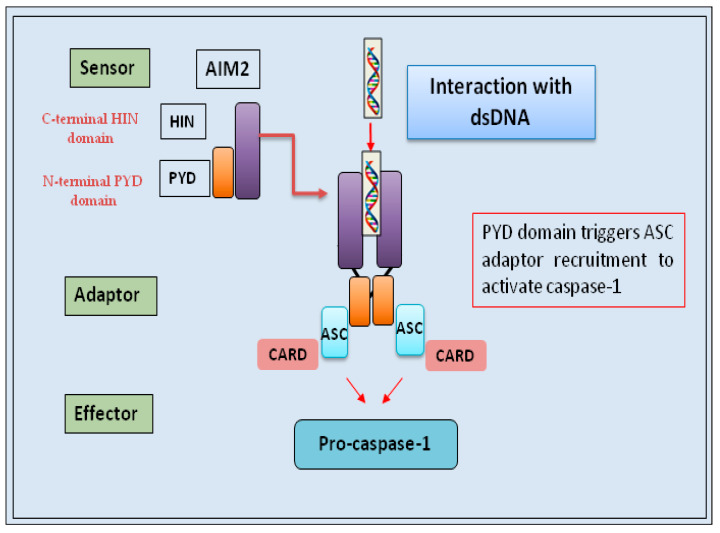
Schematic representation of AIM2 domain organization. The AIM2 inflammasome is composed of AIM2, ASC, and pro-caspase-1. AIM2 includes one pyrin domain (PYD) at the N-terminus and one or two hematopoietic interferon-inducible and nuclear (HIN) domains at the C-terminus. The pyrin and HIN domains of AIM2 form a complex and are maintained in an autoinhibitory state. The HIN domain binds to cytosolic dsDNA derived from bacteria and viruses. (AIM2: Absent in melanoma 2; CARD, C-terminal caspase recruitment domain; HIN, hematopoietic interferon-inducible and nuclear domain; PYD, pyrin domain).

Absent in melanoma 2 (AIM2) was discovered about 10 years ago. Among the already known “canonical” inflammasomes, which include the NLR family pyrin domain-containing (NLRP)1, NLRP3, NAIP/NLRC4 (NLR family apoptosis inhibitory protein/caspase activation and recruitment domain (CARD) containing (NLRC)4), and pyrin, AIM2 is currently the only one capable of detecting the presence of scattered DNA in the cytosol thanks to its ability to bind free DNA [56]. The first indication of the existence of an inflammasome capable of sensing dsDNA came from experiments evaluating virally induced inflammatory responses. Researchers discovered that inflammasome activation resulted from the simple transfection of bacterial, viral, and mammalian DNA into the cytosol [57]. It was also noted that inflammasome activation depended on ASC but not on NLRP3, a well-known inflammasome component. Furthermore, it was concluded that the formation of this inflammasome capable of sensing dsDNA also functioned independently of Toll-like receptor 9 (TLR9), another sensor of the presence of circulating DNA known to act in endolysosomes [56]. AIM2 belongs to the PYHIN family. Proteins of the mammalian PYHIN (IFI200/HIN-200) family are involved in defense against infection through recognition of foreign DNA. They have more recently been denominated the “PYHIN” family, acknowledging the defining features of one pyrin domain (PYD) at the N-terminus and one or two hematopoietic, interferon-inducible, and nuclear (HIN) domains at the C-terminus. There are four human PYHIN proteins: IFI16 (interferon-inducible protein 16) [58], MNDA (myeloid nuclear differentiation antigen) [59], AIM2 [60,61], and IFIX (interferon-inducible protein X) [61,62]. AIM2 binds cytosolic DNA via its HIN domain and initiates inflammasome formation via its pyrin domain. The PYHIN family also includes p204 in mice. AIM2 was initially discovered as an interferon-inducible tumor suppressor [60] but was later identified as a cytosolic double-stranded DNA (dsDNA) sensor that can assemble into an inflammasome with ASC and pro-caspase-1 [63,64,65,66]. ASC (apoptosis-associated granule-shaped protein containing a CARD) is critical for inflammasome assembly, which occurs using its N-terminal pyrin (PYD) domain. In the case of AIM2, it binds to dsDNA via its C-terminal HIN domain, releasing the N-terminal PYD, allowing it to interact with ASC. Following this interaction, ASC recruits pro-caspase-1, forming the AIM2 inflammasome. In 2009, four groups of researchers independently identified AIM2 as a molecule capable of sensing the cytosolic presence of dsDNA that was able to form inflammasomes with ASC, activate caspase-1, and ultimately lead to maturation of the inactive form of IL-1β or cellular apoptosis [63,64,65,66]. Building on these findings, the researchers employed two parameters like those that had allowed the identification of AIM2 to identify other molecules involved in inflammasome formation. The parameters were the search for proteins that exhibit both DNA-binding domains and the PYD for homotypic interaction with ASC [64,65,66] and, secondly, the search for proteins whose transcription is regulated by IFN-β [63]. Many proteins of the PYHIN family met both criteria, but, surprisingly, AIM2 was found to be the only one that interacts with ASC [64] and is present exclusively in the cytoplasm [65]. AIM2 has been shown to bind preferentially to DNA rather than RNA, and with greater affinity to dsDNA than to single-stranded DNA (ssDNA) [63,66]. Furthermore, the dsDNA recognized by AIM2 must possess a specific sequence, since the sequence poly(dA:dT) has been shown to bind to AIM2 and induce the formation of the ASC-binding site, known as the “ASC spot,” in an AIM2-dependent manner [64,65]. It was subsequently established that the C-terminal HIN domain of AIM2 is responsible for interaction with dsDNA, while the N-terminal PYD can interact with ASC [64,65]. An interesting finding was that, although a specific sequence is not required for the activation of the AIM2 inflammasome, the DNA length is a determining factor and consists of approximately 80 base pairs for an optimal AIM2 response [67] (for details on the AIM2 structure, see Figure 3).

## 6. Mechanisms of AIM2 Inflammasome Assembly

It follows, therefore, that the core structure of the AIM2 inflammasome is well known and was the first inflammasome structure to be evaluated and examined at the atomic level. On these assumptions, the AIM2 structure has served as a model through which to understand how other inflammasomes assemble and activate. The components of the AIM2 inflammasome do not use a simple stoichiometric model for assembly but rather act through the formation of nucleated filaments originating from molecules upstream of the assembly interactions. AIM2 recognizes both intracellular DNA originating from the cell itself and circulating cell-free DNA (cfDNA) from the extracellular space, which primarily enters cells via endocytosis and phagocytosis. Extracellular cfDNA cannot autonomously and automatically activate AIM2. AIM2 specifically recognizes dsDNA, as it is a cytosolic sensor; extracellular cfDNA must be internalized by the cell and enter the cytosol before it can bind to the HIN domain of AIM2 and trigger AIM2 assembly.

**Figure 4:**
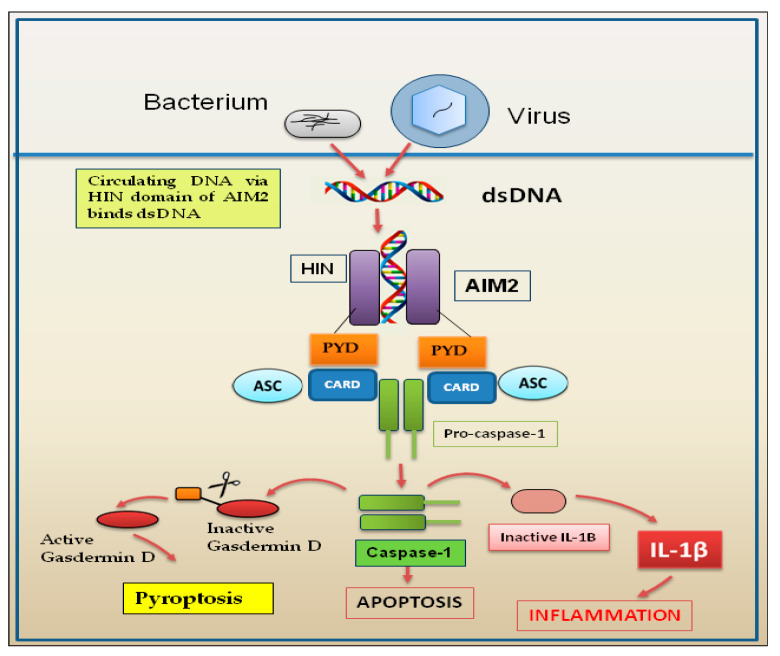
Model of AIM2 inflammasome activation. Double-stranded DNA released from bacteria and viruses binds to AIM2, inducing assembly of the AIM2 inflammasome that consists of AIM2, ASC, and pro-caspase-1. Activated caspase-1 then cleaves pro-interleukin-1β and gasdermin to mediate inflammation, pyroptosis, and apoptosis. (AIM2: Absent in melanoma 2).

The first step is the binding of AIM2 to dsDNA via the HIN domain; this binding determines the activation of AIM2 through the loss of an auto-inhibitory state or through conformational changes that lead to the oligomerization of AIM2 [67,68]. The binding and assembly mechanism of the AIM2 inflammasome follows a precise molecular sequence: the first step involves the detection of circulating DNA via the C-terminal HIN domain of AIM2, which binds dsDNA [68,69]. In the absence of stimuli, AIM2 is in a state of self-inhibition in which its PYD and HIN domains interact with each other. Binding to dsDNA displaces the PYD (pyrin domain), freeing it to interact. The free PYD of AIM2 then interacts with the PYD of an ASC-recruiting adaptor protein, causing ASC polymerization [30]. AIM2, importantly, acts as a nucleus for the polymerization of ASC, which self-assembles into long helical filaments known as “ASC specks.” The inactive pro-caspase-1 zymogen forms are subsequently recruited into these multimolecular complexes via the CARD-CARD interaction and made active by cleavage and autoproteolysis into heterodimers consisting of the two p10-p20 subunits [28] (the AIM2 assembly is reported in Figure 4). The fate of this caspase activation may be the triggering of apoptosis, pyroptosis, or chronic inflammation [70,71].

## 7. Molecular Factors Involved in AIM2 Activation in Autoimmunity

In eukaryotes, self-DNA is physiologically constrained in the nucleus and mitochondria, thus preventing the uncontrolled induction of pro-inflammatory pathways by cytoplasmic DNA-sensing mechanisms [72]. The detection of cytoplasmic DNA has been observed following various situations of altered cellular balance caused by pathogenic agents, DNA damage, aberrant cellular mechanisms involving chromatin reorganization, mitochondrial alterations, and ineffective demolitions of senescent cells [73]. Such accumulations of cytoplasmic DNA have been identified as probable triggers of inflammatory reactions in various tumor cell lines [74], as well as in tissues obtained from patients with chronic inflammatory diseases, as in the case of psoriatic lesions; often not only genomic DNA is found but also duplex RNA/DNA [75]. As has already been demonstrated for glandular epithelial cells in patients with SjD, in psoriasis keratinocytes are able to release abundant amounts of pro-inflammatory cytokines such as IL-1β, which accumulates in inflamed psoriatic skin lesions; this mechanism seems to involve a free DNA-dependent assembly and activation of the AIM2 inflammasome [76]. These findings have led to the consideration that the release of inflammatory cytokines by keratinocytes in psoriatic lesions is a determining factor for the activation of AIM2 [77]. It remains to be clarified whether the keratinocytes present in psoriatic lesions present an intrinsic upregulation of AIM2, since there are not yet sufficient experimental data deriving from primary cultures of psoriatic keratinocytes. Data collected show that the phenomenon of the presence of cytoplasmic DNA may be correlated with inflammation, based on an anomalous or altered behavior of DNases such as DNase2 and TREX1/DNase3 [78]. A close correlation between the development of an inflammatory condition and the presence of cell-free DNA in the cytoplasm has been widely demonstrated [79]. DNA derived from host cells in the cytosol may be related to the presence of DNA from damaged neighboring cells or may result from defects in the degradation and removal processes of DNA itself. A correlation between AIM2 assembly and autoimmune diseases, characterized by chronic inflammatory conditions, has been demonstrated in various diseases. In RA, AIM2 is associated with synovitis, vascular changes, cartilage destruction, and bone loss. Cytoplasmic dsDNA accumulation is one of the factors triggering the fibroblast-mediated inflammatory response in RA [80]. The initiation of inflammatory responses seems to involve, among other mechanisms, intracellular activation of AIM2, consequently enhancing the detection of cytoplasmic dsDNA and corroborating the inflammatory process in the synovium [78]. Analyzing the mRNA and protein levels of AIM2 and its downstream protein ASC, they were found to be altered in RA [78], with a higher or lower serum expression of ASC and AIM2, respectively, in RA patients compared to healthy subjects [78]. Furthermore, ASC and AIM2 expression are positively correlated with erythrocyte sedimentation rate (ESR) and C reactive protein (CRP) levels, suggesting that AIM2 is involved in the inflammatory pathogenesis of RA [81]. Furthermore, since AIM2 belongs to the genes whose transcription depends on interferon-gamma (IFN-γ) levels, and IFN-γ is certainly involved in AIM2 activation, regulates the production of various cytokines such as the release of IL-1β, IL-18, IL-6, and TNF-α, and appears to be crucial in the process of cell death (pyroptosis) [82], these data highlight the role played by AIM2 in the chronic inflammation that characterizes RA. A positive feedback mechanism is established in which chronic inflammation continuously activates fibroblasts, which in turn release large amounts of pro-inflammatory factors and cytokines [83]. The experimental efficacy of AIM2 gene silencing in RA fibroblasts has also been demonstrated, resulting in an inhibition of their proliferation without preventing their migration and apoptosis [81]. Furthermore, some studies have demonstrated the anti-inflammatory efficacy of myricetin (MYR), which seems to act precisely by reducing the gene and protein expression of AIM2 in RA patient fibroblasts [84]. Kassem et al. have demonstrated [85] that MYR can protect DNA from damage and cells from oxidative stress; therefore, the protective effect of MYR may be due to a reduction in dsDNA in the cytoplasm by indirectly inhibiting the activation of the AIM2 inflammasome. In psoriasis, for example, IL-1β release has been detected following AIM2 activation by the presence of cytosolic DNA in skin cells [86]. In systemic lupus erythematosus (SLE), increased AIM2 expression in macrophages has been reported, and this upregulation appears to be related to patients gender, since an increase has been detected only in males; conversely, female SLE patients show a decrease in AIM2 expression levels concomitant with an increase in anti-dsDNA autoantibody titers [87]. Dysregulation of type I IFN signaling is known in SLE [88,89]. Recent studies have demonstrated a correlation between this dysregulation and the anomalous formation of the AIM2 inflammasome. This correlation appears to be determined by both excessive production of IFN-β [90] and IFN-α, which modulates AIM2 levels in murine models [91]. Reduced levels of AIM2 within immune cells, correlated with a concomitant overproduction of IFN-β, have been described in mouse strains predisposed to the development of SLE [90]. Further studies have indicated a role for B-cell-activating factor (BAFF), highly expressed in circulating CD3+ T cells and in the serum of SLE patients, in the reduction in AIM2 expression [92]. In addition, the DNA-dependent activation of the AIM2 inflammasome is also widely implicated in a wide range of inflammatory diseases for which an autoimmune etiology has often been identified, including chronic kidney diseases, metabolic diseases, and neurodegenerative diseases [93,94].

## 8. AIM2 Activation in Sjögren’s Disease

### 8.1. AIM2 Activation in SjD Salivary Glands

In pSjD, the AIM2 inflammasome is characteristically activated within SGECs, triggering autoimmune responses [95]. Elevated constitutive expression of the AIM2, NLRP3, and ASC/PYCARD genes has been found [51]. This process is driven by the accumulation of damaged cytoplasmic DNA and the reduction or alteration of DNase1 activity, found predominantly in ductal cells, which appears to lead to excessive IL-1β production [95]. In particular, the elevated AIM2 inflammasome activity in SGECs from pSjD patients could be due to a malfunctioning of DNase1 leading to an accumulation of circulating cell-free DNA (cfDNA) [95]. In addition, pSjD patients who have developed MALT-NHL, or who are at high risk of developing MALT-NHL, present high serum levels of cfDNA, and the SGs are characterized by the presence of extranuclear DNA in the form of accumulations localized, predominantly, between the striated ducts and lymphocytic foci [51]. The presence of this extracellular DNA appears to be a potent stimulus for AIM2 activation when it enters the striated ducts. Consequently, AIM2 activation in SjD appears to involve salivary gland epithelial cells as the main player, which, as demonstrated by various authors, displays a persistently active intrinsic inflammatory state [19]. A recent study demonstrated that AIM2, ASC, and even caspase-1 and IL-18 levels were increased in the saliva of patients with pSjD compared to salivary levels in healthy subjects. The number of AIM2-ASC speck cells was also elevated in SGEC from patients with SjD [96]. The activation of AIM2 could be explained based on some clinical findings in patients with SjD. These patients are, in fact, characterized by the presence of serum antinuclear autoantibodies (ANA), in addition to the classically SjD-associated anti-Ro/SSA and anti-La/SSB antibodies [97]. As reported, high levels of cell-free DNA have been found in the serum [98]. Furthermore, the presence of circulating DNA seems to be crucial in the production of type 1 interferons (IFN-1) by glandular epithelial cells. These molecules would then be released into the bloodstream of patients with SjD [99]. Based on these findings, it seems likely that circulating DNA detection pathways, whether this DNA derives from cellular damage or from pathogens, could play a key role in the activation of inflammasomes. Following the entry of circulating DNA into the cytoplasm of glandular cells, various cytosolic pattern recognition receptors (PRRs) may be activated, such as toll-like 9 (TLR9) [100], AIM2 [95], and cyclic GMP-AMP synthase (cGAS) [101]. The binding of these receptors appears to be specific for the type of circulating DNA, as DNA deriving from cellular self-damage activates AIM2 and cGAS [102], while TLR9 binds more specifically to viral DNA [103]. Experimental data collected in recent years clearly demonstrate the fundamental role played by glandular epithelia in triggering and, above all, in maintaining an inflammatory state that tends to become chronic in SjD [63]. These findings have been corroborated by experiments performed on primary cultures of SGECs derived from minor salivary glands of SjD patients that demonstrated an abnormal state of activation of epithelial cells that appears closely correlated with the tissue and systemic characteristics of the disease [17,21]. Furthermore, analyzing the transcriptome of SGEC cells derived from SjD patients at various stages of disease progression, they showed gene alterations in various inflammatory pathways implicated in signaling modulation or the activation of transduction cascades, which appeared to be more severely altered in patients with higher levels of inflammation [17,21]. Among the aberrations found, some concern the mechanisms of inflammasome activation, as demonstrated and supported by the constitutive activation of caspase-1 and the elevated synthesis of IL-1β in SGEC cells of SjD patients [51]. Furthermore, an increased expression of the P2X7 receptor correlated with inflammasome activation was also observed in the salivary glands of patients [47]. The state of inflammasome activation in the salivary epithelium of patients remains only partially understood. It is now widely accepted by the scientific world that epithelial cells of the salivary glands of SjD patients are not simple spectators but actors in the triggering and maintenance of a chronic inflammatory state [2,17,19,20,21,53], which often involves the activation of signal transduction pathways mediated by the activation of NF-κB and by an elevated production of IL-1β [104,105]. These epithelial cells, and especially those of the ducts, present, in addition, a cell-autonomous activation of the AIM2 inflammasome and an altered activation of the ASC assembly dependent pyroptosis pathways; these anomalous activations would be at the basis of a constitutive activation of caspase-1 and of the production of IL-1β observed in these SGECs from SjD patients [51]. These experimental data were confirmed by the analysis of salivary biopsies from SjD patients, which confirmed an overexpression of the AIM2 inflammasome (but not of NLRP3), together with the proteins ASC/PYCARD and IL-1β. In addition, very recent data report an altered expression of the AIM2-related protein IFI16 (interferon-gamma inducible protein 16) in the form of cytoplasmic aggregates in the ductal cells of SjD patients, so much so that this protein has been considered an autoantigen associated with SjD [106]. The mechanism of AIM2 activation in SjD has also been further elucidated, and seems to be due to the appearance of endogenous or foreign DNA in the cytosol, where it is perceived by the cell as a destabilizing signal [107]. Using experimental models of cellular stress induction that have led to altered DNA replication mechanisms, a close correlation has been demonstrated between cytoplasmic DNA accumulations and AIM2 activation in the ductal epithelium of SjD patients, likely driven by aberrant DNase activity. The main culprit in AIM2 activation seems to be the damaged double-stranded DNA of genomic origin, although further investigations are underway to better define the role of foreign DNA, such as viral DNA. An association between the amount of damaged DNA accumulating within the cytoplasm and the degree of inflammation in the salivary glands of SjD patients, assessed in terms of lymphocytic infiltrates, has also been demonstrated. The reasons that make the DNA of glandular epithelial cells of SjD patients so unstable are, however, not fully clarified; an association between in situ oxidative processes [108,109] and the development of MALT-NHL was, however, identified. The role of impaired DNase1 in the formation of cytoplasmic DNA clumps has been further explored, demonstrating a reduction in DNase1 activity in ductal cells in pSjD SGs. Although this finding needs further experimental confirmation, it has been shown that in vitro activation of normal SGECs by pro-inflammatory molecules is correlated with a down-regulation of DNase1 expression. This observation is not exclusive to pSjD, but low DNase1 expression has also been detected in renal and thyroid epithelium of patients with lupus and in autoimmune thyroid diseases [110,111]. In this context, a distinction must be made between serum and intracellular DNase1. Secreted DNase1 has as its main function the degradation of circulating DNA derived from cell death or neutrophil activity [112], and, in patients with SLE or pSjD, it has an altered activity that leads to an ineffective degradation of circulating DNA, causing inflammatory reactions [100,113]. Intracellular DNase1 is responsible for DNA digestion in apoptotic and necrotic cells [114]; in particular, the malfunctioning of intracellular DNase1 in renal epithelial cells is, for example, related to the development of severe lupus nephritis and is characterized by massive deposits of intracellular DNA [110]. A distinct role for intracellular and serum DNase1 has also been demonstrated in SGs, where DNase 1 appears to limit the onset of intrinsic inflammatory reactions in ductal cells, and this also appears to occur for AIM2 activation; indeed, in vitro silencing of DNase1 in ductal cells results in activation of the AIM2 inflammasome [95]. Although further experimental confirmation is needed, it is hypothesized that DNase1 may perform its activity alone or in collaboration with other DNases, given the abundance of these enzymes in renal, intestinal, or salivary epithelia [115]. The cellular aberrations observed in the ductal epithelium of SjD patients resemble those physiologically occurring during cellular senescence, with loss of nuclear membrane integrity and intracellular DNA accumulation and the activation of NF-κB- and IL-1-dependent pro-inflammatory pathways [115]. The chronic and cell-autonomous inflammatory state that predominantly affects the ductal epithelial cells of patients with SjD could therefore be explained by a deficient DNase1 activity.

### 8.2. AIM2 Activation in SjD Lacrimal Glands

The lacrimal gland is a tubuloacinar exocrine gland that produces tears, composed essentially of water, proteins, and electrolytes [116], and is one of the main organs affected by pSjD. The lacrimal gland epithelium is composed of ductal, acinar, and myoepithelial cells (MEC). The MECs, specifically, arrange themselves around the acinar cells, which constitute the glandular secretory epithelium itself, and thanks to the contraction of the myofibrils (containing alpha-SMA), they facilitate secretion. The MECs, moreover, facilitate the secretion of the extracellular matrix to form the basement membrane of the acini and regulate the exchanges between secretory cells and stroma, which are essential in the repair processes of tissue damage [117]. Altered AIM2 gene expression has been demonstrated in cultured MECs derived from the lacrimal gland in a mouse model of pSjD [118]. In lacrimal MECs, homologous gDNA activates both the AIM2 inflammasome and cGAS-STING (Stimulator of Interferon Genes) pathways, reducing MECs contractility and inducing the secretion of pro-inflammatory cytokines that cause cell death. Studies performed using in vitro cultures of cells derived from lacrimal gland epithelium have refined the understanding of the relationship between the sensing of endogenous intracellular DNA and the initiation and perpetuation of pSjD. Indeed, recent studies have demonstrated that internalized endogenous DNA causes inflammation and cellular dysfunction in the lacrimal gland MECs [102]. The cGAS protein binds to DNA, exploiting electrical charges, via its nucleotidyltransferase domain. This binding mechanism is valid for any type of DNA in an undifferentiated manner. The AIM2 binding site, however, has a high affinity for nucleotides A and T [119]. This is supported by experiments conducted in vitro, which have shown that using a positive control represented by a poly AnT, an oligonucleotide containing A and T, a strong activation of the pathway mediated by the activation of AIM2 and a simultaneous reduction in the contractility of the MECs are obtained. In vivo, the situation could be more complex than the system appropriately miniaturized in vitro, due to the great variability of pathological intracellular or extracellular DNA. Endogenous extracellular DNA, for example, is a complex mixture of DNA combined with proteins, but it can also be present in extracellular vesicles, circulating mitochondrial DNA, and DNA fragments [79]. These heterogeneous DNAs are still able to activate the inflammatory pathways mediated by AIM2 or cGAS [120]. Therefore, further investigations are inevitably needed to clarify whether there are differences in the triggering of inflammation depending on the type of triggering DNA. Data on inflammasome activation in lacrimal glands in SjD are limited and essentially demonstrate the role of exogenous DNA in chronic inflammation [102]. These are either neutrophil-derived exogenous DNA, including increased release of neutrophil extracellular traps (NETs) composed of DNA, histones, and antimicrobial proteins [121], the results of impaired clearance of post-apoptosis cellular debris or ineffective DNase1 [51,99]. When neutrophils release the DNA during inflammation (e.g., in NETs), it acts as an exogenous-like signal (DAMP) that is targeted by exogenous agents like DNase. An important role, however, is played by endogenous DNA, which appears to be able to activate lacrimal gland MECs following tissue damage or chronic inflammation [122]. This appears to be related to a decrease in the efficacy of DNase2b and the overexpression of multiple endogenous DNA-activated signaling molecules in the lacrimal gland. These recent data indicate a reduced ability to clear cytoplasmic DNA from lacrimal gland cells in pSjD. Among the molecules capable of modulating the mechanisms of free endogenous DNA detection, inhibitors of AIM2 or the STING protein seem to have the promised prospects, appearing to effectively attenuate inflammation during in vitro experimental procedures. To confirm this efficacy, it is essential to further investigate the mechanisms of identification and removal of endogenous or exogenous DNA and to conduct in vivo efficacy studies that will confirm the use of these molecules as potential therapeutic agents in SjD. In addition, the inflammasome activation, involving caspase-1 and IL-1β, appears to be dependent on the level of IFN-β in lacrimal gland MECs. In fact, when cells are stimulated with endogenous DNA, only when IFN-β reaches a certain threshold value does IL-1β secretion increase. Type I IFN activates the transcription of genes dependent on it only following binding to its type I IFN receptor (IFNAR) [123] and only after it has reached values that potentiate positive feedback responsible for the exacerbation of the inflammatory response [102]. Researchers subsequently wondered whether this inflammasome activation originates exclusively from MECs in the lacrimal glands, where these cells undoubtedly play a key role in promoting the regular secretion of tear fluid. Molecular communication between MECs and acinar epithelial cells leading to the activation of AIM2 and STING has been observed. This cellular communication probably also involves infiltrating immune cells and the paracrine regulation carried out by cGAMP [120]. This paracrine communication mechanism occurs via the purinergic receptor P2X7 [124], expressed in the lacrimal gland [125]. However, the data obtained are related to genetically modified experimental mouse models that develop a disease like SjD, but with limitations. Currently, in vivo data confirming these mechanisms in SjD are lacking, although there is strong evidence that MECs respond to endogenous genomic DNA released following damage, inducing activation of inflammatory mechanisms with activation of AIM2 and STING inflammasomes and apoptosis, involving epithelial and glandular immune cells. Table 1 summarizes the experimental evidence related to the activation of NLRP3 and AIM2 in SjD.

## 9. Therapeutic Modulation of the Inflammasome in SjD

Currently, therapeutic options for the treatment of SjD are mainly aimed at alleviating symptoms such as dry eyes and mouth, monitoring any systemic manifestations with the aim of improving quality of life [3]. In most patients, the drugs used are common antirheumatic drugs or glucocorticoids, which have the effect of reducing and controlling the chronic inflammation that damages the structure of the affected organs. These drugs halt or reduce the progression of the disease but have little effect on the prevalent symptoms such as dryness and chronic fatigue [3,4,5,6]. Over the past two decades, molecules targeting specific components of the immune system have been evaluated to identify new therapeutic avenues for SjD [3]. Given the important role played by B lymphocytes [126] in the pathogenesis of SjD, several biologics acting either against B-cell cellular targets (such as rituximab and epratuzumab) or against the cytokine-soluble B-cell-activating factor (BAFF) (such as belimumab) have been tested, but these drugs have failed to live up to expectations, showing poor efficacy in SjD [127,128,129]. Inhibition of the NLRP3 inflammasome could be a viable strategy for the treatment of pSjD. A recent study by Zhou et al. evaluated the use of the MCC950 molecule to attenuate the activation of NLRP3, using an experimental animal model represented by a strain of NOD mice, commonly used as a model of SjD. MCC950 is a highly specific inhibitor that acts by preventing the assembly of NLRP3, as deduced from several studies both in vitro and in vivo and conducted on murine models of different inflammatory diseases [130]. Its targeted efficacy is determined by the fact that it prevents the formation and activation of NLRP3 mediated by the ATPase and does not show consequences on other types of inflammasomes, such as AIM2, NLRC4, and NLRP1 [131]. The use of the specific inhibitor has advantages over other inhibitory techniques such as gene silencing. However, to date, the study of the effects of NLRP3 activation, both in cells infiltrating the SGs of patients with SjD and in SGECs, has not clarified the specific mechanisms by which the NLRP3 inflammasome operates in SjD.

Iguratimod, a compound widely used as a synthetic antirheumatic drug, has also been evaluated as an indirect inhibitor of NLRP3 [132]. Iguratimod (also known as IGU or T-614) is a modified small molecule that can inhibit NF-κB activation by interfering with its translocation from the cytoplasm to the nucleus. Studies were performed both on an experimental murine model of SjD and on primary cultures of human SGECs treated with INF-α. The inhibitory effect of T-614 on the NLRP3 inflammasome is not new but has already been demonstrated in other inflammatory pathologies such as bleomycin-induced pulmonary fibrosis, in which this molecule is able to reduce the EMT process and the activation of the NLRP3 inflammasome [133]. Hou et al. demonstrated the efficacy of T-614 in severe acute pancreatitis through the inhibition of NLRP3 inflammation and the NF-κB pathway [134]. The study conducted by Liu et al. confirmed the efficacy of T-614 in reducing inflammation in the glands of mice used as an experimental model of SjD by acting on the activation of NLRP3. In murine glands, this molecule reduced lymphocytic infiltration and lowered the levels of IL-6, IL-18, and IL-1β. Furthermore, treatment with T-614 determined a reduction in the activity of IFN-α, NLRP3, caspase-1, and ASC in SGEC [132]. The possibility of interfering with the activation of NLRP3 was also evaluated based on the use of natural compounds. He et al. reported that ruscogenin ameliorated pSjD by suppressing NLRP3 inflammasome activation [135]. Ruscogenin is an active component of *Ophiopogon japonicus*, with strong anti-inflammatory properties [136]. Ruscogenin appears to interfere with the activation of NLRP3 in cases of cerebral ischemia by acting on the blood–brain barrier [136]. The focus of this study was on the P2X7R receptor, considered to be a key receptor in inflammation; this receptor has been correlated with the maturation of pro-inflammatory molecules such as IL-1β. On the other hand, it has been verified that P2X7R causes inflammatory reactions worsening the prognosis, specifically through a strong activation of the NLRP3 inflammasome [52]. The effect of ruscogenin would be directly on the expression of P2X7R, NLRP3, caspase-1, and IL-1β in murine models of SjD. This effect seems to occur in a dose-dependent manner and places ruscogenin among the potentially therapeutic molecules in SjD. Encouraging results were obtained by transfection of plasmids containing NLRP3 genes into acinar cells of SGs. These important investigations have demonstrated an anti-inflammatory role of Ruscogenin associated by an anti-apoptotic effect in acinar cells through the negative modulation of the NLRP3 inflammasome [137].

Xu et al. have reported that the Huoxue Jiedu Recipe (HXJDR), a kind of traditional Chinese medicine, diminishes the damage in SGs by inhibiting the NLRP3 inflammasome [137] acting on mitochondrial dysregulation, which represents one of the leading causes in SjD progression.

Another natural compound has demonstrated some efficacy in SjD by acting on the regulation of NLRP3. This compound includes total glucosides extracted from white peony, specifically peoniflorin (PF), the main active ingredient of these glucosides, which possess antioxidant and anti-inflammatory effects [138]. Using NOD mice, a well-known experimental model of SjD, PF alleviated SjD-like symptoms. PF-treated mice showed a significant reduction in NLRP3 and caspase-1 in SGs, with a simultaneous decrease in serum levels of IL-1β and IL-18, compared to controls. A clear inhibition of NLRP3 induced by PF was also demonstrated. PF inhibits NLRP3 activation through activation of the Nrf2/HO-1 pathway in SGECs. The nuclear factor erythroid 2-related factor 2 (Nrf2) is a transcription factor responsible for the regulation of cellular redox balance; heme oxygenase-1 (HO-1) is one of the genes regulated through Nrf2 [139]. PF therefore appears to inhibit NLRP3 inflammasome activation through regulation of the Nrf2/HO-1 axis in SGs of SjD mice, and this could be used for therapeutic purposes [138].

Finally, Ianalumab (VAY736) appears to have shown some efficacy in the treatment of SjD [140]. It is a novel human monoclonal antibody that acts on B cells, causing their depletion through antibody-dependent cellular cytotoxicity via blockade of the BAFF receptor (BAFF-R). This interrupts the transduction cascade triggered by binding to this receptor, halting the maturation, proliferation, and survival of B cells. It is hypothesized that it also prevents the reactivation of residual pathogenic B cell clones in the target tissue, which could offer a long-term efficacy of Ianalumab treatment. Although a direct effect on NLRP3 has not yet been evaluated, this antibody could be considered for future studies, given its activity on B lymphocytes (Table 2 summarizes the experimental and clinical evidence reported in paragraph 9).

## 10. Conclusions

**Figure 5:**
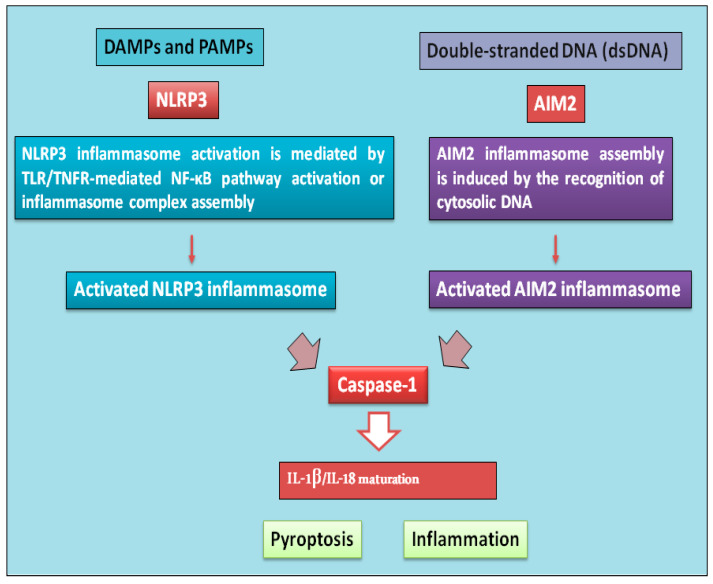
Schematic representation of interaction of NLRP3 and AIM2 inflammasomes in SjD. NLRP3 and AIM2 present distinct triggers: AIM2 senses double-stranded DNA while NLRP3 responds to diverse molecular patterns such as DAMPs or PAMPs. Assembly of NLRP3 or AIM2 leads to the maturation of caspase-1 and functionalization of IL-1β and IL-18. AIM2 (Absent in melanoma 2); NLRP3 (NLR family pyrin domain containing 3); PAMPs (Pathogen-Associated Molecular Patterns); DAMPs (damage-associated molecular patterns).

In conclusion, this review highlights the regulatory role of NLRP3 and AIM2 and the molecules involved in their activation in the chronic inflammation that characterizes SjD. The manuscript summarizes recent data highlighting how dysregulated assembly and activation of NLRP3 and AIM2 may play a key role in the pathogenesis of SjD and in the abnormal activation of the immune response underlying this autoimmune disease. The correlation between NLRP3 and AIM2 activation in SjD is reported in Figure 5.

We hope this review will encourage researchers to further explore this field of investigation to clarify inflammasome targets and activating molecules in the ongoing search for new therapies.

## Figures and Tables

**Table 1:** Details of activation signals, expression sites, and associated diseases of the NLRP3 and AIM2 inflammasomes in SjD. AIM2 (Absent in melanoma 2); DAMPs (damage-associated molecular patterns); NLRP3 (NLR family pyrin domain containing 3); PAMPs (Pathogen-Associated Molecular Patterns); PBMC (peripheral blood mononuclear cells); SGEC (salivary gland epithelial cells).

Inflammasome	Cell Type	Tissue Distribution	Trigger Signals	Associated Tissue Phenotype	State of Disease	Refs.
NLRP3	Epithelial cells, immune cells: dendritic cells, macrophages, and CD4+ T lymphocytes	Serous acini of salivary and lachrymal glands	Single-stranded RNA viruses—Specific autoantibodies (anti-Ro/SSA)—ROS (from tissutal oxidative stress)	Sjögren’s disease	Xerostomia and xerophthalmia	[22]
NLRP3	PBMS	Serum	DAMPS, PAMPS, nucleic acids released following tissue injury	Sjögren’s disease	Gland dysfunction, apoptosis, non-Hodgkin’s lymphoma	[47,48]
P2X7 receptor (P2X7 R)-NLRP3 inflammasome complex	Epithelial cells, PBMC	Labial salivary glands	Degree of inflammatory response	Primary Sjögren’s disease anti-Ro/SSA positivity and correlated with FS of <1	Gland dysfunction, apoptosis, non-Hodgkin’s lymphoma	[47,51]
NLRP3	PBMC, circulating monocytes	Salivary glands	DNA accumulations	Primary Sjögren’s disease, FS of <1	Gland dysfunction, apoptosis, non-Hodgkin’s lymphoma	[51]
AIM2	SGECs	Salivary glands: ductal cells	Damaged cytoplasmic DNA, alteration of DNase1 activity,	Primary Sjögren’s disease	Severe inflammatory state, MALT lymphoma	[95,97,106,107]
AIM2	Myoepithelial cells	Lacrimal gland	Aberrant self-DNA	Primary Sjögren’s disease (mouse model)	Release of pro-inflammatory cytokines, impairment of the contractility of myoepithelial cells, apoptosis	[118]
AIM2	Myoepithelial cells	Lacrimal gland	Endogenous DNA	Primary Sjögren’s disease	Inflammation and cellular dysfunction in the lacrimal gland MECs	[102]
AIM2	Myoepithelial cells	Lacrimal gland	Endogenous extracellular DNA	Primary Sjögren’s disease	Severe chronic inflammation, alteration of secretion of tear fluid	[79,102,120,121,122,123,124,125]

**Table 2:** Therapeutic modulation of NLRP3 in SjD animal models or patients.

Drug	Mechanism	Model Used	Refs
MCC950	It prevents the assembly and activation of NLRP3	NOD mice—SjD model	[130]
Iguratimod	Inhibits NF-κB activation	NOD mice—SjD model ; primary cultures of human SGECs	[131,132,133,134]
Ruscogenin	Inhibits the NLRP3 activation in cases of cerebral ischemia by acting on the blood-brain barrier	NOD mice—SjD model	[135,136]
Huoxue Jiedu Recipe	Diminishes the damage in SGs by inhibiting the NLRP3 inflammasome	NOD/Ltj mice; SjD patients	[137]
Peoniflorin	Inhibition of NLRP3 through activation of the Nrf2/HO-1 pathway in SGECs	NOD mice—SjD model	[138,139]
Ianalumab	Human monoclonal antibody that acts on B cells, causing their depletion through antibody-dependent cellular cytotoxicity via blockade of the BAFF receptor	Patients with SjD; efficacy through week 52 and a favorable safety profile up to two years of follow-up.	[140]

## Data Availability

No new data were created or analyzed in this study. Data sharing is not applicable to this article.
